# Differentiation between Wild-Type Group A Rotaviruses and Vaccine Strains in Cases of Suspected Horizontal Transmission and Adverse Events Following Vaccination

**DOI:** 10.3390/v14081670

**Published:** 2022-07-29

**Authors:** Sonja Jacobsen, Sandra Niendorf, Roswitha Lorenz, C.-Thomas Bock, Andreas Mas Marques

**Affiliations:** 1 Unit Gastroenteritis and Hepatitis Viruses and Enteroviruses, Department of Infectious Diseases, Robert Koch Institute, Seestraße 10, 13353 Berlin, Germany; jacobsens@rki.de (S.J.); niendorfs@rki.de (S.N.); roswitha.lorenz@freenet.de (R.L.); bockc@rki.de (C.-T.B.); 2 Consultant Laboratory for Rotaviruses, Robert Koch Institute, Seestraße 10, 13353 Berlin, Germany; 3 Institute of Tropical Medicine, University of Tuebingen, Wilhelmstraße 27, 72074 Tuebingen, Germany

**Keywords:** rotavirus A, acute gastroenteritis, vaccination, virus shedding, diagnostic workflow, molecular diagnostics, adverse events, co-infections, horizontal transmission

## Abstract

Human group A rotaviruses (RVA) are important enteric pathogens, as they are a leading cause of acute gastroenteritis (AGE) in children worldwide. Since 2013, the German Standing Committee on vaccination recommended the routine rotavirus vaccination for infants in Germany. While vaccination has significantly decreased RVA cases and worldwide mortality, in some cases, infants can develop acute gastroenteritis as an adverse reaction after immunization with an attenuated live vaccine. Pediatricians, as well as clinicians and diagnostic laboratories, contacted the Consultant Laboratory for Rotaviruses and inquired whether cases of RVA-positive AGE after vaccination were associated with vaccine or with wild-type RVA strains. A testing algorithm based on distinguishing PCRs and confirmative sequencing was designed, tested, and applied. Diagnostic samples from 68 vaccinated children and six cases where horizontal transmission was suspected were investigated in this study. Using a combination of real-time PCR, fragment-length analysis of amplicons from multiplex PCRs and confirmative sequencing, vaccine-like virus was detected in 46 samples and wild-type RVA was detected in 6 samples. Three mixed infections of vaccine and wild-type RVA were detectable, no RVA genome was found in 19 samples. High viral loads (>1.0 × 10^7^ copies/g stool) were measured in most RVA-positive samples. Furthermore, information on co-infections with other AGE pathogens in the vaccinated study population was of interest. A commercial multiplex PCR and in-house PCRs revealed three co-infections of vaccinated infants with bacteria (two samples with *Clostridioides difficile* and one sample with enteropathogenic *E. coli*) and six co-infections with norovirus in a subset of the samples. Human astrovirus was detected in one sample, with suspected horizontal transmission. The cases of suspected horizontal transmission of vaccine RVA strains could not be confirmed, as they either involved wild-type RVA or were RVA negative. This study shows that RVA-positive AGE after vaccination is not necessarily associated with the vaccine strain and provides a reliable workflow to distinguish RVA vaccine strains from wild-type strains.

## 1. Introduction

Group A rotaviruses (RVA) are a leading cause of acute gastroenteritis (AGE) worldwide, particularly in children. A high percentage of children have been infected with RVA by three years of age, but older children and adults can also be affected [1]. RVA infections can cause severe diarrheal disease and dehydration in infants and young children and are highly associated with mortality in low-income countries [2,3]. It was estimated that 128,500 deaths caused by diarrhea were attributable to rotavirus infection among children younger than 5 years of age in 2016 [4].

Rotaviruses are classified as the genus *Rotavirus* in the family *Reoviridae*. The genome of 11 segments encodes for six viral structural proteins, VP1 to VP4 and VP6 to VP7, as well as six non-structural proteins (NSP1 to NSP6) [5]. Classification of RVA is standardized for all 11 genome segments, including a binary G+P-genotyping system based on VP7 (G type) and VP4 (P type) [6]. Currently, 42 different VP7 G types and 58 different VP4 P-types have been accepted by the Rotavirus Classification Working Group, and at least 16 G-types and 19 VP4 P-types have been identified for RVA strains infecting humans [7,8]. A small number of RVA genotype combinations circulate globally in the human population at higher frequencies, including combinations of G1, G3, G4, G9, G12 with P[8], and G2 with P[4]. Additionally, in some regions, P[6] is relevant in different genotype constellations [9].

The German Standing Committee on Vaccination (STIKO) has recommended the rotavirus vaccination for infants in Germany since 2013 [10]. Vaccination mimics natural RVA infections and has been identified as a major strategy to decrease the burden associated with severe and fatal rotavirus-induced diarrhea [11,12]. So far, two commercial vaccines have been licensed in Germany since 2006. The monovalent Rotarix^®^ vaccine (RV1) contains a live attenuated human G1P1A[8] RVA strain. RotaTeq^®^ (RV5) is a pentavalent bovine–human vaccine of five reassortant bovine strains containing genes that express outer capsid proteins of five common circulating human RVA strains (G1, G2, G3, G4, and P[8]), together with the antigens originating from the bovine RVA strain used as a vector (G6 and P7[5]). The vaccines RV5 and RV1 are administered orally to infants in two or three doses, respectively [10]. RVA vaccinations, as well as previous natural RVA infections [13], do not induce full protection against re-infection with wild-type RVA strains. However, AGE hospitalization rates significantly decreased after vaccination, as did episodes of severe AGE [10,14,15,16].

Adverse reactions can follow after RVA immunization in infants, especially AGE. Shedding of both live vaccines has been detected in stool samples for several days or weeks [17,18]. Shed vaccine strains can be transmitted to unvaccinated children, adults, and the elderly, where they can cause AGE [19,20,21,22]. The methods in the present study were developed to answer inquiries from pediatricians, as well as clinicians and diagnostic laboratories, regarding whether cases of AGE after vaccination were associated with vaccine strains or were due to coincidental infection with wild-type RVA strains. Molecular methods which reliably discriminate between vaccine-like strains and common wild-type RVA are useful for monitoring the rate of occurrence and clinical relevance of vaccine strains in symptomatic pediatric RVA infections in the relevant age group worldwide [23,24].

Therefore, a PCR-based algorithm including sensitive and highly specific PCRs was developed to identify RVA infections and to distinguish wild-type RVA strains from vaccine-like strains in human specimens, even in mixed infections.

## 2. Materials and Methods

### 2.1. Samples

A total of 72 stool samples, one cerebrospinal fluid sample (CSF), and one serum sample were investigated in this study, sent in for diagnostic characterization during the years 2009 to 2019. Sixty-eight out of seventy-four samples were from RV-vaccinated children with AGE, and six samples were from cases with suspected horizontal transmission. Stool samples were diluted 1:10 in phosphate-buffered saline before RNA extraction.

### 2.2. Design of the Workflow for Molecular Diagnostics and Discrimination between RVA Wild-Type and RVA Vaccine-like Strains

RNA was extracted from 140 µL of stool suspension, serum, or CSF using a QIAcube device (Qiagen, Hilden, Germany) with the QIAamp Viral RNA Mini Kit (Qiagen, Hilden, Germany) and eluted in a total volume of 60 µL AVE (supplied elution buffer).

The first step of the RVA-discriminating workflow was the screening of samples for RVA genome with a sensitive and highly specific real-time RT-PCR method, as described previously [25]. Only RVA-positive samples were subjected to further analysis for the differentiation of wild-type RVA and vaccine-derived RVA.

**Figure 1:**
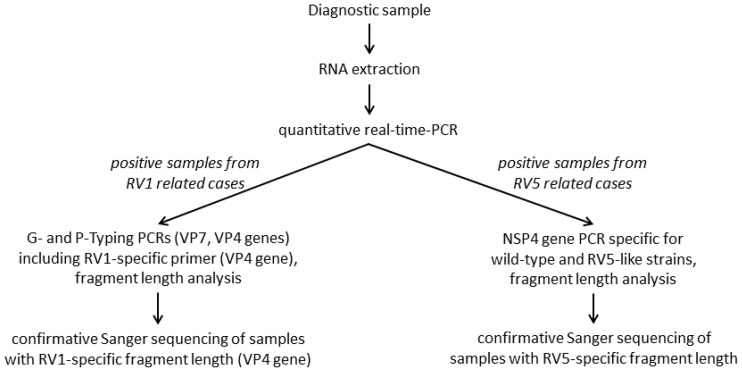
Workflow of the molecular differentiation of wild-type RVA and vaccine-like strains from RV1 and RV5 related cases.

Two highly specific and sensitive RT-PCR assay systems were implemented in the workflow of the RVA discrimination algorithm to identify wild-type RVA strains versus RV5-, or versus RV1 vaccine-like strains (Figure 1). The PCRs were applied according to known vaccination with RV5 or RV1. If no information on vaccination was available, both PCR systems were applied.

### 2.3. Discrimination of Wild-Type RVA and RV5

Discrimination between human wild-type RVA and RV5 was achieved using multiplex nested RT-PCR with primer sets located in the NSP4 gene of the RVA genome, producing amplicons of 119 bp to 122 bp in the second PCR round for RVA wild-type viruses and 197 bp for RV5 vaccine-like strains. First set of outer primers (Table 1): RV5-like (RoA71; RoA74); wild-type strains: (RoA61; RoA64c, RoA64d; RoA64e; RoA64f). Second PCR round: RV5-like (RoA72; RoA73), wild-type strains (RoA63a; RoA63b; RoA63c). All primers were purchased from TIB Molbiol (Berlin, Germany). After denaturation of RNA at 95 °C for 1 min, RT-PCR was performed with 5 µL of RNA in a total reaction volume of 12.5 µL with 240 nmol/l primers using a One-step RT-PCR kit (Qiagen, Hilden, Germany). Reverse transcription (RT)-reaction was carried out at 50 °C for 30 min and 15 min at 95 °C, followed by 30 cycles at 94 °C for 30 s, 42 °C for 30 s, 30 s at 72 °C, and a final elongation step at 72 °C for 5 min. The second PCR round was as follows: PCR products were diluted in water (1:20) and 1 µL (of the dilution) was added to the second PCR round in a reaction volume of 12.5 µL using HotStarTaq Master Mix Kit (Qiagen, Hilden, Germany) and 240 nmol/l primers. PCR conditions were as follows: 15 min at 95 °C, 30 cycles of 30 s at 94 °C, 30 s at 42 °C, 30 s at 72 °C, and a final elongation step at 72 °C for 5 min. PCR amplicons were visualized by agarose gel electrophoresis and the PCR results were confirmed by direct sequencing.

The validation of the workflow included testing of the specificity, sensitivity, and reproducibility in intra- and inter-assay comparison of three independent runs. Selected pre-characterized samples of RVA-positive patients (RVA wild-type virus strains) and RV5 vaccine were used to test the system. First, RVA genome in the samples was quantified by a previously described real-time RT-PCR [25]. After creating serial 1:10 dilutions of the quantified samples, the detection limit was estimated with endpoint titration and quantification of the endpoint dilution with the real time RT-PCR system. Determination of the detection limit was achieved using virus dilutions at the estimated detection limit, as well as dilutions 10-fold higher, 3-fold higher, 0.3-fold lower, and 0.1-fold lower than the estimated detection limit. The detection limit of the multiplex RV5/wild-type RT-PCR was defined as the lowest concentration where at least 95% of 24 samples tested positive (8 replicates tested in 3 independent runs). The determined detection limit was 600 RNA copies/reaction (1.3 × 10^6^ copies/g stool) of wild-type virus and 60 RNA copies/reaction (1.3 × 10^5^ copies/g stool) of RV5. Additionally, 100% reproducibility was found via intra- and inter-assay comparison of samples with the RV5 and a G1P[8] wild-type strain.

The specificity of the assay was tested with samples from patients with AGE which were known to be positive for human norovirus (3 samples) or positive for sapovirus (3 samples) or positive for three enteroviruses (echovirus 18, coxsackie B5 virus, and coxsackie A9 virus). All of these samples were negative.

### 2.4. Discrimination of Wild-Type RVA and RV1

Differentiation of wild-type RVA and RV1 was performed via a multiplex nested RT-PCR reaction using VP4-genotyping primers based on a published P typing assay [26] complemented with a new RV1-specific primer (published primer set [27], added RV1-specific sense primer, Table 1: RoA83; all primers from TIB Molbiol (Berlin, Germany). The different genotype-specific amplicon sizes of RVA P-types ([27], Table 1) can be determined by agarose gel electrophoresis.

In order to distinguish between wild-type P[8]- and vaccine-derived strains more reliably, especially in mixed infections, capillary fragment-length analysis was used to increase the overall specificity of amplicon size calling (ABI 3500xL Dx device and GeneMapper 5.0 software: Applied Biosystems, Forster City, CA, USA) [26]. Fragments of 246 bp for RV1-like strain and 224 bp for RVA wild-type strains could be distinguished reliably by two sharply distinct peaks. For confirmation, the RV1-like fragments were sequenced directly (Sanger) with PCR primers.

### 2.5. Detecting Co-Infections with Other AGE Pathogens

Samples from 2009 to 2015 that were PCR positive for RVA vaccine-like strains (RV1, RV5) and negative for wild-type RVA viruses were additionally investigated for the presence of other gastroenteritis pathogens using the multiplex PCR system GastroFinder™ SMART 17 FAST (PathoFinder B.V., Maastricht, Netherlands), detecting: *Campylobacter jejuni*, *Campylobacter spp.*, *Clostridioides difficile* toxin A/B, *Escherichia coli* O157:H7, *Salmonella spp.*, Shiga toxin-producing *E. coli* (STEC), *Shigella spp.*, *Yersinia enterocolitica*, *Aeromonas spp.*, *Giardia lamblia*, *Entamoeba histolytica*, *Cryptosporidium spp.*, *Dientamoeba fragilis*, adenovirus (F40/41), astrovirus, RVA, norovirus (NV) (GI/GII/GIV), and in-house PCR assays for NV GI and GII, sapovirus (SaV), and all human astrovirus (HAstV) [28,29].

Samples from 2016 onwards were tested for NV, SaV, and HAstV using in-house PCR assays as previously described [30].

## 3. Results

### 3.1. Differentiation between Wild-Type RVA and RV5-like Strains

The differentiation of RV5-like strains and RVA wild-type virus strains was based on a multiplex reverse-transcription (RT) nested-PCR of the NSP4 gene, resulting in different fragment lengths for RV5-like and wild-type strains in both PCR rounds (Table 1).

This assay was used to test diagnostic samples for RV5-like strains using fragment-length analysis (Table 2). RV5-like PCR fragments were sequenced for verification. No discrepancies between fragment length analysis and sequencing were found. Mixed infections could be successfully sequenced using RV5- or wild-type specific primers, respectively.

### 3.2. Differentiation between Wild-Type RVA and RV1-like Strains

For specific detection of RV1-like strains, a well described and widely used P-typing method [26,27] was modified by addition of a primer with a higher melting temperature specific to RV1-like strains (RoA83, Table 1). Fragment length analysis was used to distinguish wild-type P[8] and RV1-like P[8] strains, followed by confirmative sequencing (Table 2). By sequencing all diagnostic samples, specificity of 100% for distinguishing wild-type and RV1-like strains was found. The RoA83 primer was also included in P typing for molecular surveillance of more than 5000 samples (data not shown), and RV1-positive results were verified via sequencing.

### 3.3. Patients

In this study, 74 patients with AGE were included: 68 vaccinees with suspected adverse reactions after vaccination, and 6 relatives with suspected horizontal transmission (Table 2). The median age of vaccinees was three months (range 1–10 months, two patients without data) and the rate of male and female vaccinees in this study was 56% and 43%, respectively (no gender was communicated in one case). Thirty-six children were vaccinated with RV1 and thirty-two were vaccinated with RV5.

**Table 2:** Patients and results of samples from 2009 to 2019.

Patient No.	Sampling Year	Age [mo.]	Gender	Vaccine Used	d.p.v.	RV1/RV5 Detected	Wild-Type RVA Detected	Other AGE Pathogen Detected	ID
1	2009	2	Female	RV1	5	+	−	NV	
2	2009	5	Male	RV5	n.d.	−	−	NV	
3	2009	5	Female	RV5	34	−	−	Not tested	
4	2009	4	Female	RV5	>30	−	−	Not tested	
5	2009	4	Male	RV5	n.d.	+	−	−	SCID
6	2009	6	Male	RV1	76	−	−	Not tested	
7	2010	4	Male	RV1	6	+	−	−	SCID
8	2010	2	Female	RV5	7	+	−	−	
9	2011	2	Female	RV1	16	+	−	−	
10	2011	6	Male	RV1	6	+	G1P[8]	C. diff.	
11	2011	10	Male	RV1	n.d.	−	G9P[8]	Not tested	Unspecif.
12	2011	n.d.	Male	RV5	n.d.	−	−	Not tested	
13	2011	n.d.	N.d.	RV5	n.d.	+	−	−	
14	2012	2	Female	RV5	7	+	−	−	
15	2013	5	Male	RV5	n.d.	+	−	−	SCID
16	2013	2	Female	RV1	5	−	G9P[8]	Not tested	
17	2013	3	Female	RV5	7	+	−	−	
18	2014	6	Male	RV5	n.d.	+	−	−	
19	2014	3	Male	RV1	7	+	−	−	
20	2014	7	Female	RV5	6	−	−	Not tested	
21	2014	1	Male	RV1	14	−	−	Not tested	
22	2014	4	Female	RV5	n.d.	−	−	Not tested	
23	2014	3	Female	RV1	n.d.	+	−	−	
24	2014	4	Female	RV1	n.d.	−	−	Not tested	
25	2014	4	Female	RV1	29	−	−	Not tested	
26	2014	3	Female	RV1	n.d.	+	−	−	
27	2014	3	Female	RV1	n.d.	+	−	−	
28	2015	2	Male	RV5	n.d.	+	−	C. diff.	
29	2015	5	Male	RV5	n.d.	+	−	−	
30	2015	2	Female	RV5	3	+	G9P[8]	EPEC	
31	2015	12	Female	Not vaccinated ^§^	-	−	G9P[8]	Not tested	
32	2015	5	Male	RV5	n.d.	+	−	−	
33	2015	4	Female	RV5	n.d.	−	G3Px	Not tested	
34	2015	8	Male	RV5	n.d.	+	−	−	
35	2015	2	Male	RV1	9	+	−	−	
36	2016	9	Male	RV1	200	+	−	−	SCID
37	2016	4	Male	RV5	3	−	−	−	
38	2016	2	Male	RV1	10	+	−	NV	
39	2016	3	Male	RV5	7	−	−	−	Suspected
40	2016	5	Male	RV1	33	+	−	−	
41	2016	1	Male	RV1	17 *	+	−	−	
42	2016	4	Male	RV5	34	−	−	−	
43	2016	3	Female	RV1	34	+	−	−	
44	2016	3	Male	RV1	23	+	−	−	
45	2016	2	Female	RV1	17	+	−	NV	
46	2017	3	Female	RV1	30 *	−	−	−	
47	2017	6	Male	RV1	n.d.	−	−	−	
48	2017	5	Male	RV5	5	+	−	−	
49	2017	2	Male	RV1	8	+	−	−	
50	2017	2	Female	RV5	15	+	−	−	
51	2017	2	Male	RV5	9	+	−	−	
52	2017	7	Female	RV5	60 *	+	−	−	SCID
53	2017	765	Female	Not vacc. °	n.d.	−	G2P[4]	−	
54	2017	3	Male	RV5	49	−	−	−	
55	2017	2	Male	RV5	6	+	−	−	
56	2017	2	Female	RV1	12	−	−	−	
57	2017	2	Female	RV5	35	+	−	−	
58	2017	2	Male	RV1	17	+	−	−	
59	2017	2	Male	RV1	15	+	−	−	
60	2018	4	Female	RV1	9	−	−	NV	
61	2018	2	Male	RV1	12	+	G3P[8]	−	
62	2018	352	Female	Not vacc. ^§§^	n.d.	−	G3P[8]	−	
63	2018	19	Female	Not vacc. ^§§^	n.d.	−	G3P[8]	−	
64	2018	92	Female	Not vacc. ^§§^	n.d.	−	G3P[8]	HAstV	
65	2018	2	Male	RV5	10	+	−	−	
66	2018	3	Female	RV5	45	+	−	−	
67	2018	6	Female	RV1	72	+	−	−	Suspected
68	2019	4	Male	RV1	11	+	−	−	
69	2019	2	Female	RV1	7	−	G2P[4]	−	
70	2019	2	Female	RV1	12	+	−	−	
71	2019	7	Male	RV5	195*	+	−	−	
72	2019	3	Male	RV1	36	+	−	−	
73	2019	<1	Male	Not vacc. °	28	-	−	−	
74	2019	4	Male	RV1	47	+	−	NV	

Not vacc.—not vaccinated, d.p.v.—days post vaccination, ID—immunodeficiency, n.d.—no data were present, * approximation (only month of vaccination available), § sibling to vaccinated patient 30, §§ relatives to vaccinated patient 62, ° no sample from suspected source received.

### 3.4. Shedding of Vaccine and Wild-Type Strains

Vaccine strains were detected in 46 of 68 vaccinees, but not in patients with suspected horizontal transmission (Table 3). Three vaccinees were positive for both vaccine and wild-type virus. Mixed infections were positive for RV1 and wild-type G1P[8], RV1 and G3P[8], and RV5 and G9P[8], respectively. Four vaccinees were negative for vaccine strains but positive for wild-type virus: two for G9P[8], one for G2P[4], and one for G3 (P not typeable).

**Figure 2:**
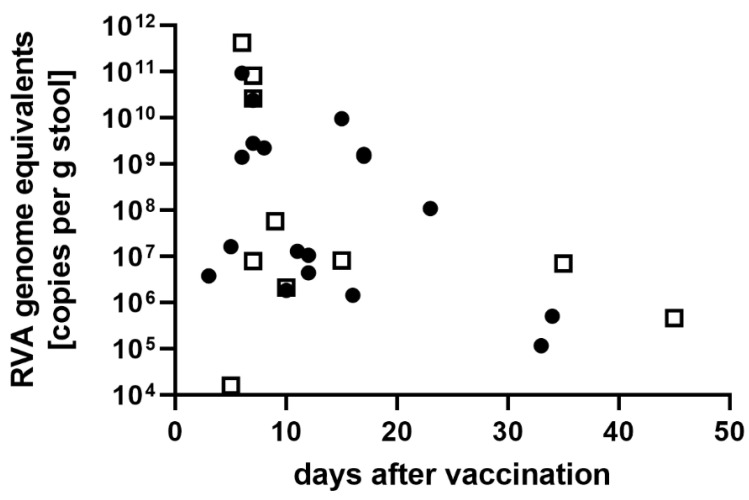
RVA genome equivalents (viral load) detected in samples from immune-competent vaccinees (no known immune deficiencies). Closed circles: samples from RV1-vaccinated children, open squares: samples from RV5-vaccinated children.

Regarding virus shedding after vaccination, a correlation analysis was performed with detected viral loads (RVA genome copies/g stool) and the time interval between vaccination and collection of the stool. Information on the exact date of vaccination and the sample collection date was available for 28 RVA-positive vaccinees (Figure 2), excluding samples from patients with known immunodeficiencies (prolonged shedding) and patients with mixed infection (vaccine strain and wild-type strain). RV1 vaccinated children had virus loads between 1.2 × 10^5^ to 3.0 × 10^12^ copies/g stool with a median of 1.1 × 10^8^ copies/g stool. RV5 vaccinated children showed copy numbers from 1.6 × 10^4^ to 4.2 × 10^11^ copies/g stool with a median of 8.0 × 10^6^ copies/g stool. The earliest day of sampling was three days after vaccination. The highest copy numbers were found in the first week after the last vaccination with RV1 (3.8 × 10^6^ to 9.4 × 10^10^, median 2.1 × 10^9^ copies/g stool) and RV5 (1.6 × 10^4^ to 4.2 × 10^11^, median 2.7 × 10^10^ copies/g stool). After seven days, the median for RV1 and RV5 was 1.2 × 10^7^ copies/g stool (range 1.2 × 10^5^ to 9.7 × 10^9^ copies/g stool) and 7.0 × 10^6^ copies/g stool (range 4.6 × 10^5^ to 5.8 × 10^7^ copies/g stool), respectively. RVA genome could be detected up to 45 days after vaccination.

### 3.5. RVA Shedding in Patients with Immunodeficiencies

Five children had severe combined immunodeficiencies (SCID), whereas immunodeficiency disease was unspecified in one case and suspected in two cases (Table 2). Four of these infants were vaccinated with RV5 and another four with RV1. The course of infection was described in detail for patient 36 [31]. This RV1-vaccinated patient, who suffered from SCID, persistently shed RV1 and only became RVA negative six months after hematopoietic stem cell transplantation [31].

Detected viral loads of vaccinees with SCID were in the range of 1.4 × 10^9^ copies/g stool to 3.0 × 10^12^ copies/g stool. In the five vaccinated children with SCID, vaccine-derived RVA was detectable, while the child with unspecified immunodeficiency disease was positive for wild-type G9P[8].

### 3.6. Suspected Horizontal Transmission

Suspected horizontal transmission of vaccine strains was also analyzed in the study group (Table 3). Two samples were from siblings: an unvaccinated girl of 12 months and her sister of 2 months of age, who was vaccinated with RV5 (patients 30 and 31, Table 2). Both children were positive for RVA, with viral loads of 3.9 × 10^11^ copies/g stool for the vaccinated child and 1.9 × 10^11^ copies/g stool for the unvaccinated sibling. In both children, wild-type RVA G9P[8] was detectable. Additionally, the vaccinated child was positive for vaccine-derived RVA and EPEC.

Another case of suspected horizontal transmission was that of patient 53, where the grandchild (no sample received) was vaccinated and the grandmother, who had an autoimmune disease, developed diarrhea and acute renal failure. A wild-type G2P[4] RVA was detected, but no vaccine strain was found.

In the case of patient 61, horizontal transmission after vaccination to his mother and two siblings (patients 62, 63 and 64, Table 2) was suspected. Both RV1 vaccine strain and wild-type G3P[8] strain were found in the sample of patient 62. However, the samples of all relatives contained only the wild-type G3P[8] strain.

Finally, horizontal transmission was suspected in patient 73, a premature infant with necrotizing enterocolitis where the mother (no sample received) had contact with an RV1-vaccinated child. No RVA whatsoever was detected in the sample of the premature infant.

### 3.7. Screening for Co-Infection with Other AGE Pathogens

To analyze if vaccine strains were the only possible factor in AGE after vaccination, the 22 specimens that tested positive for RVA vaccine strains from 2009 to 2015 and 1 RVA-negative sample were also screened for co-infections with other causative agents of AGE using a commercial kit for gastrointestinal bacterial and viral pathogens (Table 2). Furthermore, all 37 samples from 2016 to 2019 were tested for norovirus (NV), human astroviruses (HAstV), and sapovirus (SaV). Co-infections of vaccine strains with NV were detected in four samples. Two NV-positive samples were negative for any RVA strain, and HAstV was detected in a sample positive for a RVA wild-type strain. No SaV was found. In three samples, human pathogenic bacterial genome was present, showing two samples with *Clostridioides difficile* (C. diff) and one with enteropathogenic *E. coli* (EPEC) (Table 2).

## 4. Discussion

In this study, an algorithm was developed and applied for diagnostic differentiation between vaccine-like RVA viruses and wild-type RVA strains. A panel of 68 samples from children with AGE who were vaccinated with either RV1 or RV5 was analyzed. In six additional cases, horizontal transmission of vaccine strains was suspected. Shedding of RVA was detectable in the majority of the samples. It is well known that vaccination with live attenuated RVA vaccines comes along with virus shedding. In this regard, a review of different studies estimated virus shedding of RVA to be approximately 10% (RV5) to 50% (RV1) of vaccine recipients when analyzed by ELISA [32]. Asymptomatic shedding of RV5 was detected in vaccinated children for up to 84 days after the third immunization dose [18,33]. In children with immune deficiency, shedding was detectable for a prolonged time [23,31,34,35]. One study demonstrated that the peak of virus shedding after administration of the first dose occurred between day 4 and day 7, with highest viral loads at day 6 and 7 [17]. It was reported that, after RV1 vaccination, the mean titer of virus shedding was 1.7 × 10^9^ copies/g stool, and for RV5, the mean titer was 2.6 × 10^7^ copies/g stool [17], which is lower than the virus loads in the present study. In this study with symptomatic vaccinees, the highest viral loads were also found within the first week after vaccination, peaking at day 7. However, the median viral load of RV5 strains was one order of magnitude higher than RV1 and more than two orders of magnitude higher than the previously determined peak of viral load for RV5 strains in healthy vaccinees at 6–7 days [17]. Due to the limited number of samples in the present study, this finding should be interpreted carefully. Moreover, while shedding of high viral loads of wild-type RVA has been associated with AGE and low viral loads with asymptomatic infection [36,37], healthy vaccinees shed high amounts of attenuated vaccine strains [17]. This is in line with a recently published study, in which no correlation between shedding of RVA vaccine strain and symptoms of AGE was observed [38]. Thus, shedding of high viral loads of RVA vaccine strains does not have a clear correlation with symptomatic infection, in contrast with RVA wild-type infection. Due to attenuation, vaccine strains can replicate at high levels without causing symptoms. A recent study showed no significant difference concerning the duration and amount of shedding of RV5 strains between asymptomatic and symptomatic vaccinees [24].

The situation is different with primary immune deficiencies. Five infants with SCID shed vaccine-like RVA while one child with unspecified immune deficiency disease suffered from wild-type virus infection. SCID often has only been diagnosed when a more severe infection or generally high morbidity became obvious in infants. However, live attenuated vaccines are of risk for children with SCID [39]. Shedding of vaccine strains at high viral loads can persist for months until immunocompetence has been established by hematopoietic stem cell transplantation or other therapies [31,35,40]. The WHO and STIKO emphasize the contra-indication of RVA vaccination in SCID patients [10,11]. In Germany, detection of SCID was added to the scope of general examinations of neonates by the Federal Joint Committee (G-BA) in July of 2019, which will help to prevent further cases of severe disease in vaccinees with SCID.

As duration of shedding and viral load of vaccine strains do not clearly correlate with disease in immunocompetent vaccinees, the detection of unspecified RVA or vaccine strain in particular is not a fully reliable indicator for the etiological agent. Therefore, screening for other possible pathogens should be considered. Co-infection of RVA with other gastroenteritis pathogens has been described. Depending on the published study design, RVA was detected as co-infection with, e.g., adenovirus, norovirus, astrovirus, sapovirus, bocavirus, *Clostridioides difficile*, enterotoxigenic *Escherichia coli*, *Shigella*, *Campylobacter jejuni*, *Giardia*, and *Entamoeba histolytica* [41,42,43,44]. In the present study, co-infections of RVA with other AGE pathogens was demonstrable in 8 out of 51 tested samples (16%). Mixed infection of RVA vaccine and wild-type strains was found in three of the 50 RVA-positive vaccinees (6%). Thus, differentiation of vaccine and wild-type strains is needed to identify co-incidental infection with wild-type RVA as a possible etiological agent of AGE, and complements screening for pathogens.

For diagnostic differentiation, a set of RT-PCRs specific for the vaccines RV1 or RV5 were designed and applied, as shown in the present study. These assays have been used for more than 10 years. They rely on a basic RT-PCR setup with fragment-length analysis for both vaccine strains, and do not include real-time PCR, sequencing, or digestion by restriction enzymes for differentiation, and therefore differ from previously published studies with respect to methodology [20,23,24,45]. The sensitivity for detection of mixed infections with vaccine and wild-type strains is higher than Sanger sequencing, still distinguishing up to three orders of magnitude difference in vaccine and wild-type copy numbers. Discrimination of the strains can be achieved via gel electrophoresis with acceptable specificity. However, due to higher-resolution fragment-length analysis, using a capillary sequencer for the highly multiplexed P typing assay (RV1) is helpful to exclude false-positive signals that may result from multiplex PCR of nucleic extracts from stool samples. Additionally, sequencing confirms if the detected vaccine-like strains from fragment-length analysis are actually vaccine derived or (very rarely) just closely related wild-types, and identifies possible genetic drift after longer periods of shedding. However, confirmative sequencing of RV1-like strains revealed no case of mistyping by fragment-length analysis. This is in line with the finding that the vast majority of circulating P[8] strains are of lineage III [46], and therefore differ significantly from the RV1 strain (lineage I). The same applies to the RV5 vaccine strains, all of which contain the NSP4 gene originating from a bovine rotavirus. Thus, confirmative sequencing might be added as additional layer of information, but is not needed in every setting.

Specific detection of wild-type and vaccine strains was also used in the cases of suspected horizontal transmission of vaccine strains by relatives. If RVA was found in samples from relatives, it was wild-type RVA, whereas vaccinees had mixed infections with wild-type and vaccine strains. While no horizontal transmission of vaccine strains was detected in the present study, vaccine strains can be transmitted and may cause symptoms. A possible dose dependency was discussed in the case of an unvaccinated two-year-old sibling with AGE, who was infected with RV1 after handling the stool-discharged diaper of the younger sibling that had been vaccinated [47].

In the present study, RV1 and RV5 vaccine strains and wild-type strains with common genotypes were detected (VP7: G1, G2, G3 and G9, VP4: P[4] and P[8]). With the implementation of live attenuated RVA vaccines, surveillance and monitoring studies started to trace the impact of RV immunization to the prevalence of common RVA genotypes and the emergence of immune escape RVA strains [20,48,49]. Worldwide, there is great diversity in wild-type RVA strains. This diversity is mainly influenced by several mechanisms: accumulation of point mutations, reassortment, direct transmission of animal strains into humans, and gene rearrangement into coding or non-coding regions [50]. Due to differences in the diversity of RVA genotypes in developing and developed countries, it is important to monitor genotype variation worldwide to detect vaccine-reassortant strains [45,50,51,52]. The specific detection of RV1 strains described in the present study, enabled by addition of primer RoA83 to a published P typing assay, is also being used for molecular surveillance [27].

With regard to the introduction of rotavirus vaccines such as Rotavac^®^ and Rotasiil^®^, further vaccine development [53] and the impact of different vaccines on the herd immunity, it is mandatory to monitor virus variability [54]. A previously published study investigated the effectiveness of mixed rotavirus vaccinations [55]. Administration of mixed vaccines will pose a challenge for discrimination between wild-type RVA and vaccine strains in the future.

Previous studies reported on different methods of discriminating RVA strains from vaccine-like strains [20,45,56,57,58]. An assay distinguished between RV1 vaccine-like strain and RVA wild-type virus targeting the NSP3 gene using multiplex RT-PCR and BspHI-endonuclease restriction-length polymorphism [20]. Another RT-PCR method discriminated between RV5 vaccine-like and RVA wild-type, targeting the NSP3 gene [56]. A previously described method is based on real-time RT-PCR technology. This assay focused on the differentiation of RV5, RV1, and wild-type RVA strains in a multiplex reaction, detecting RVA G/P types G12, G9, G4, G3, G2, P[4], P[8], and P[6], respectively. However, the authors recommended confirming RV-positive real-time PCR results with direct sequencing or next-generation sequencing [57]. Bucardo et al. applied full-genome sequencing and detected reassortants of wild-type strains with a NSP2 gene identical to the RV5 strains, which would not be detectable with assays focusing on specific other genes, including the assays presented here. To prevent reassortment with vaccine strains and reduce the risk of adverse events, non-replicating RVA vaccines could be an alternative in the future, if they prove to be at least as efficacious as current live vaccines [59,60].

Since the workflow in the present study is mainly based on nested RT-PCR techniques and fragment-length analysis, it is economical and easy to implement with basic laboratory equipment and therefore useful in any setting. It is also low budget. A capillary sequencer for fragment-length analysis and Sanger sequencing is helpful for confirmation and increased specificity, but is not required.

In conclusion, this study provides relevant data concerning the detection of RVA vaccine and wild-type strains, as well as viral loads in cases of suspected adverse reactions and horizontal transmission. An algorithm for use in differential diagnosis and molecular surveillance has been developed to discriminate reliably between RVA wild-type and RVA vaccine-like strains in patient samples using molecular methods. The emphasis was on an easy-to-follow protocol including application of common molecular methods.

The specific detection of RVA wild-type strains as likely etiological agents instead of vaccine strains in some cases added important data for differential diagnosis. It is not sufficient to apply common RVA assays to cases of AGE with suspected involvement of RVA vaccine strains. The detection of RVA should include discrimination between vaccine and wild-type strains.

## Figures and Tables

**Table 1:** Primers and probes used for algorithm of RVA discrimination.

Assay	Target	Fragment Length	Primer Name	Primer Sequence
VP4 gene (complements P typing PCR [26])	RV1-like	246 bp	RoA83	CTT GCT TTC ACC AAA TAT CA
NSP4 gene 1st PCR round	**RV5-like**	**398 bp**	**RoA71**	AAA GAT GGA TAA GCT TAC
(multiplex: RV5, wild-type)			**RoA74**	CGT GAA TGC GTT TTA GT
	*Wild type*	*451 to 452 bp*	*RoA61*	TCT GTT CCG AGA GAG C
			*RoA64c*	CTC AYC AGT YGA TCG MAC
			*RoA64d*	CTC GCC AGT TGA TYG MAC
			*RoA64e*	TAR CGT CAR CTG GTY TAG
			*RoA64f*	TAG TGT CAA CCG GTC TAG
NSP4 gene 2nd PCR round	**RV5-like**	**197 bp**	**RoA72**	ACA GCA CAT TGC ACA CG
(nested to 1st PCR round,			**RoA73**	TGC CAA TTT CAA CAA CGC
multiplex: RV5, wild type)	*Wild type*	*119-122 bp*	*RoA62b*	ACA YTA CAY AAA GCD TCA
			*RoA63a*	CCT GCT ARC KTT AAT AAT GT
			*RoA63b*	TAT CCT GCC AAC TTT AAA AGA G
			*RoA63c*	CCT GCT AGT TTC ART AAC GT

Fragment lengths and primers specific for RV5-like strains in **bold**, for wild type in *italic* font.

**Table 3:** Frequency of RVA vaccine and wild-type strains detected in the study group.

Detected RVA Strains	All Patients	Vaccinated	Not Vaccinated
**Vaccine strain**	43	43	0
**Wild-type strain**	9	4	5
**Vaccine + wild-type strain**	3	3	0
**Negative**	19	18	1
**All**	74	68	6

## Data Availability

The data presented in this study are available on request from the corresponding author.
